# Validation of a new transpulmonary thermodilution system to assess global end-diastolic volume and extravascular lung water

**DOI:** 10.1186/cc9332

**Published:** 2010-11-23

**Authors:** Karim Bendjelid, Raphael Giraud, Nils Siegenthaler, Frederic Michard

**Affiliations:** 1 Department of APSI, Geneva University Hospitals, 4 rue Gabrielle-Perret-Gentil, Genève 14-1211, Switzerland; 2 Department of Critical Care, Edwards Lifesciences, 70 route de l'Etraz, Nyon 1260, Switzerland

## Abstract

**Introduction:**

A new system has been developed to assess global end-diastolic volume (GEDV), a volumetric marker of cardiac preload, and extravascular lung water (EVLW) from a transpulmonary thermodilution curve. Our goal was to compare this new system with the system currently in clinical use.

**Methods:**

Eleven anesthetized and mechanically ventilated pigs were instrumented with a central venous catheter and a right (PulsioCath; Pulsion, Munich, Germany) and a left (VolumeView™; Edwards Lifesciences, Irvine, CA, USA) thermistor-tipped femoral arterial catheter. The right femoral catheter was used to measure GEDV and EVLW using the PiCCO_2_™ (Pulsion) method (GEDV_1 _and EVLW_1_, respectively). The left femoral catheter was used to measure the same parameters using the new VolumeView™ (Edwards Lifesciences) method (GEDV_2 _and EVLW_2_, respectively). Measurements were made during inotropic stimulation (dobutamine), during hypovolemia (bleeding), during hypervolemia (fluid overload), and after inducing acute lung injury (intravenous oleic acid).

**Results:**

One hundred and thirty-seven paired measurements were analyzed. GEDV_1 _and GEDV_2 _ranged from 701 to 1,629 ml and from 774 to 1,645 ml, respectively. GEDV_1 _and GEDV_2 _were closely correlated (*r*^2 ^= 0.79), with mean bias of -11 ± 80 ml and percentage error of 14%. EVLW_1 _and EVLW2 ranged from 507 to 2,379 ml and from 495 to 2,222 ml, respectively. EVLW_1 _and EVLW_2 _were closely correlated (*r*^2 ^= 0.97), with mean bias of -5 ± 72 ml and percentage error of 15%.

**Conclusions:**

In animals, and over a very wide range of values, a good agreement was found between the new VolumeView™ system and the PiCCO™ system to assess GEDV and EVLW.

## Introduction

Transpulmonary thermodilution (TPTD) is increasingly used for hemodynamic evaluations in critically ill patients [1-4]. After injection of a cold indicator in the superior vena cava, TPTD allows the computation of cardiac output (CO) from a TPTD curve recorded by a thermistor-tipped femoral arterial catheter [4]. Additional physiological parameters can be derived from the dilution curve, such as global end diastolic volume (GEDV), a volumetric marker of cardiac preload [5-7], and extravascular lung water (EVLW) [7-10].

The TPTD method currently in clinical use and implemented in the PiCCO™ system (Pulsion Medical Systems, Munich, Germany) is based on mathematical models described in the 1950 s [11,12]. A new and original method has recently been developed to derive GEDV and EVLW from a TPTD curve (VolumeView™; Edwards Lifesciences, Irvine, CA, USA). The aim of the present animal study was to compare the new VolumeView™ system with the PiCCO™ system, over a wide range up to extreme pathophysiological conditions.

## Materials and methods

The study was approved for the use of swine by the Institutional Animal Care and Use Committee at the Edwards Lifesciences Biological Resource Center, and all experimentation was done in accordance with the Guide for the Care and Use of Laboratory Animals (1996; ILAR, NAP, Washington, DC, USA).

Eleven anesthetized and mechanically ventilated pigs (90 to 110 kg) were studied. Animals were premedicated with intramuscular midazolam (0.5 mg/kg) and atropine (0.5 mg) and were anesthetized with an injection of propofol (1 mg/kg) followed by continuous infusion of propofol (150 μg/kg/min) and sufentanil (2.5 μg/kg/h). After tracheal intubation, pigs were mechanically ventilated in a volume-controlled mode with a FiO_2 _of 50%, a respiratory rate between 12 and 16 breaths/minute (to maintain an end-expiratory partial pressure of carbon dioxide within the normal range), a positive end-expiratory pressure of 0 cmH_2_O and a tidal volume of 10 ml/kg.

**Figure 1:**
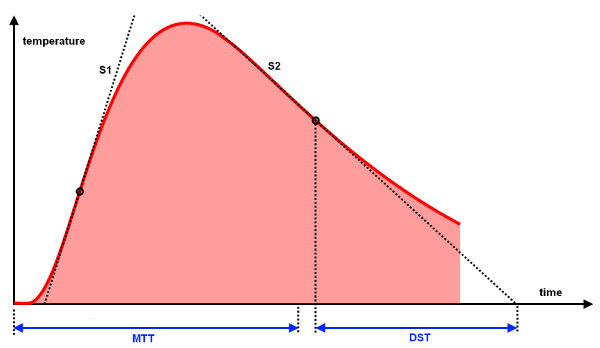
**Transpulmonary thermodilution curve**. The assessment of global end-diastolic volume (GEDV) by the PiCCO™ system is based on the mean transit time (MTt) and exponential downslope time (DSt), while the assessment of GEDV by the new VolumeView™ method is based on MTt, maximum ascending slope (S1) and maximum descending slope (S2).

All animals were instrumented with a right (PulsioCath™; Pulsion Medical Systems) and a left (VolumeView™; Edwards Lifesciences) 5F thermistor-tipped femoral arterial catheter. The correct position of femoral catheters was confirmed by radioscopy (Figure 1).

All animals were also instrumented with a pulmonary artery catheter (CCComboV™, 7.5F; Edwards Lifesciences) inserted through the right jugular vein and with a central venous catheter in the left jugular vein (Figure 1). The pulmonary artery catheter was used for continuous monitoring of CO (Vigilance II; Edwards Lifesciences) and pulmonary arterial pressures during the experimental protocol. The central venous catheter was used for cold indicator injections and for central venous pressure monitoring. Pulmonary artery pressures, continuous CO and central venous pressure data were used to guide therapy at various stages (as described below) but were not recorded nor analyzed.

### The current transpulmonary thermodilution system

The right femoral catheter was connected to a PiCCO_2_™ monitor (Pulsion Medical Systems) and used to measure CO (CO_1_), GEDV (GEDV_1_) and EVLW (EVLW_1_) using the following equations [1,7,9,10]:

$${\text{CO}}_{1}\;=\;V_{i}\left(T_{b}\;-\;T_{i}\right)k/\text{AUC}$$

where *V*_i _is the injectate volume, *T*_b _is blood temperature, *T*_i _is injectate temperature, *k *is a constant proportional to the specific weights and specific heat of blood and injectate, and AUC is the area under the TPTD curve.

$${\text{GEDV}}_{1}\;=\;{\text{CO}}_{1}\;\times \left(\text{MTt}\;-\;\text{DSt}\right)$$

where MTt is the mean transit time of the cold indicator and DSt is the exponential downslope time (Figure 1).

$${\text{EVLW}}_{1}\;=\;\left({\text{CO}}_{1}\times \;\text{MTt}\right)-\left(1.25\;\times \;{\text{GEDV}}_{1}\right)$$

The new transpulmonary thermodilution system

The left femoral catheter was connected to the EV1000™ monitor (Edwards Lifesciences) and used to measure CO (CO_2_), GEDV (GEDV_2_) and EVLW (EVLW_2_). CO was derived from the dilution curve using the same Stewart Hamilton equation:

$${\text{CO}}_{2}\;=\;V_{i}\left(T_{b}-T_{1}\right)/\text{AUC}$$

GEDV, however, was derived from a different equation as follows:

$${\text{GEDV}}_{2}\;=\;{\text{CO}}_{2}\times \;\text{MTt}\;\times f\left(\text{S}2/\text{S}1\right)$$

where S1 and S2 are respectively the maximum ascending and descending slopes of the thermodilution curve (Figure 1) and *f *is a proprietary function.

Finally, EVLW was assessed using the equation:

$${\text{EVLW}}_{2}\;=\;\left({\text{CO}}_{2}\times \text{DSt}\right)\;-\;\left(0.25\times {\text{GEDV}}_{2}\right)$$

The same cold saline bolus injected through the central venous catheter was used to compute simultaneously the two transpulmonary curves: one with the right femoral catheter PiCCO_2_™ (Pulsion Medical Systems), the other with the left femoral catheter (EV1000™; Edwards Lifesciences). The average of three bolus measurements was considered for analysis and is reported in Results.

### Experimental protocol

**Figure 2:**
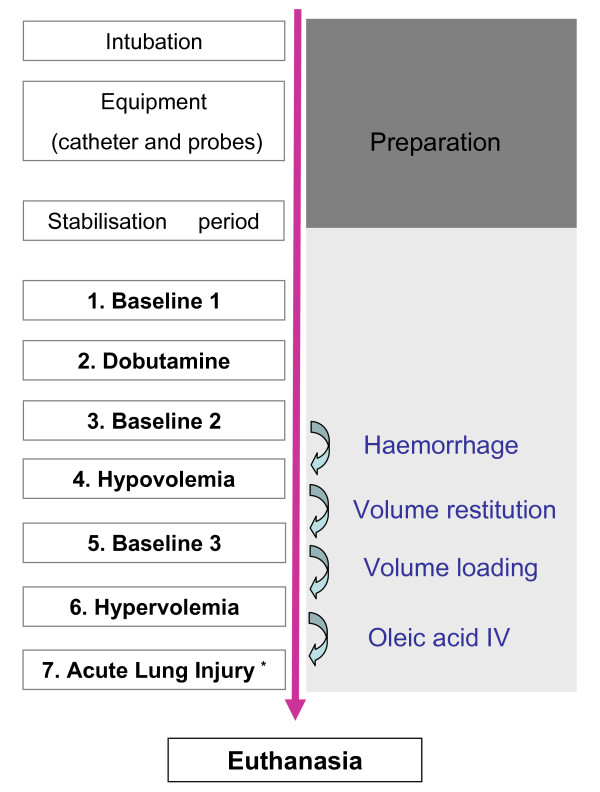
**Flow chart of the experimental protocol**. *Multiple measurements. IV, intravenous.

The experimental protocol is summarized in Figure 2. Measurements were performed: at baseline; during dobutamine infusion (DOBU, starting at 7.5 μg/kg/minute and titrated to induce a 30 to 50% increase in continuous CO); 5 minutes after stopping dobutamine infusion; after inducing hypovolemia (HYPO, controlled hemorrhage to decrease mean arterial pressure (MAP) around 50 mmHg); after blood restitution and fluid loading (2/3 blood + 1/3 serum saline); and after fluid overloading (HYPER, 75% serum saline + 25% gelatin in order to increase MAP up to 130 mmHg and/or central venous pressure up to 20 mmHg). At each stage, a 10-minute stabilization period was observed before doing the measurements. Finally, additional measurements were performed after inducing acute lung injury (ALI) by injecting intravenously oleic acid (O1383, 100 mg/kg/hour).

At this stage, several measurements were performed successively in order to capture high EVLW values. Oleic acid-induced pulmonary edema was confirmed by the occurrence of arterial hypoxemia (drop in PaO_2_/FiO_2 _and SaO_2_), a drop in the compliance of the respiratory system (increase in airway pressures while the tidal volume was maintained constant or even decreased) and lung infiltrates on chest X-ray scan (Figure 1). At this point, FiO_2 _and the positive end-expiratory pressure were respectively increased up to 100% and 15 cmH_2_O when necessary to maintain SaO_2 _> 90%. Oleic acid may induce a dramatic increase in pulmonary artery pressures and a decrease in CO (right ventricular failure). Phenylephrine and dobutamine were therefore also administered when necessary to maintain MAP >50 mmHg and continuous CO >5 l/minute as long as possible. When it was no longer possible to maintain SaO_2 _> 90% and MAP >50 mmHg, data collection was stopped and animals were sacrificed (with pentobarbital and phenytoin).

### Statistical analysis

Results are expressed as the mean ± standard deviation (SD), unless specified otherwise. Percentage errors for CO, GEDV and EVLW comparisons were calculated as twice the SD of the bias over the average CO, GEDV or EVLW value, respectively [13]. All bias, SDs, limits of agreement (2SD) and percentage errors reported in the manuscript have been corrected for multiple measurements according to the method proposed by Bland and Altman [14].

Reproducibility of TPTD measurements was assessed by calculating the standard deviation/mean ratio of triplicate measurements and is expressed as a percentage. The effect of each intervention (DOBU, HYPO, HYPER, ALI) versus the previous stage was assessed using a parametric test (paired *t *test) or nonparametric test (paired Wilcoxon test) when appropriate. Values obtained using both methods were also compared at each stage using unpaired tests (parametric or nonparametric as appropriate).

Several measurements were performed at the latest stage (ALI) in order to capture high EVLW values. At this stage, only measurements corresponding to the maximum EVLW_1 _(the reference method in the present study) have been selected for comparisons with EVLW_2_. For the linear regression analysis, however, all measurements were taken into account. *P *< 0.05 was considered statistically significant.

## Results

A total of 137 paired measurements were available for comparisons. Sixty-six paired measurements were collected from stages 1 to 6 (6 stages × 11 pigs) and 71 additional paired measurements (6.5 ± 2.1 per pig) were collected at the final lung injury stage. No data were discarded. The reproducibility of hemodynamic parameters is reported in Table 1.

**Table 1:** Reproducibility of transpulmonary thermodilution measurements

	PiCCO™ method	VolumeView™ method
Cardiac output (%)	6.3 ± 5.1	5.7 ± 4.9
Global end-diastolic volume (%)	6.8 ± 5.3	6.9 ± 5.0
Extravascular lung water (%)	5.5 ± 4.0	5.7 ± 4.2

Data presented as mean ± standard deviation.

**Figure 3:**
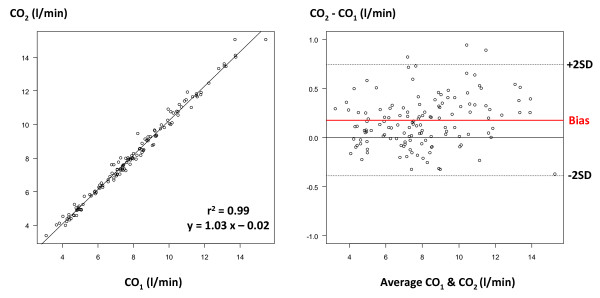
**Cardiac output comparison**. Left: correlation between cardiac output (CO) measured by the PiCCO™ system (CO_1_) and the VolumeView™ system (CO_2_). Right: Bland-Altman representation depicting the agreement between both methods. SD, standard deviation.

**Figure 4:**
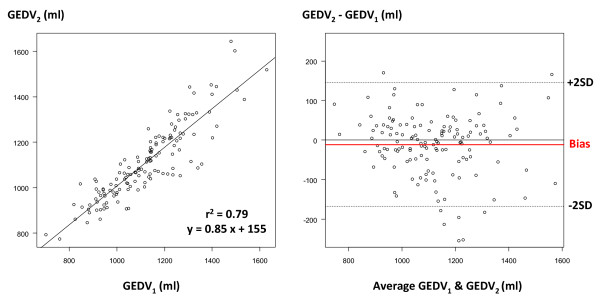
**Global end-diastolic volume comparison**. Left: correlation between global end-diastolic volume (GEDV) measured by the PiCCO™ system (GEDV_1_) and the VolumeView™ system (GEDV_2_). Right: Bland-Altman representation depicting the agreement between both methods. SD, standard deviation.

**Figure 5:**
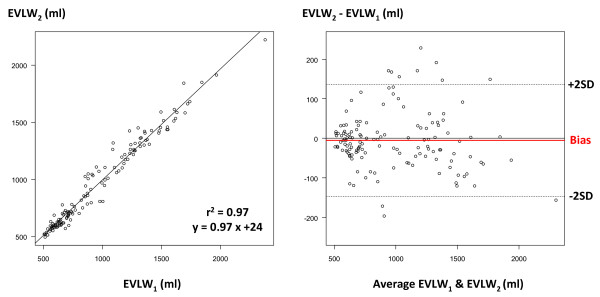
**Extravascular lung water comparison**. Left: correlation between extravascular lung water (EVLW) measured by the PiCCO™ system (EVLW_1_) and the VolumeView™ system (EVLW_2_). Right: Bland-Altman representation depicting the agreement between both methods. SD, standard deviation.

Overall, CO_1 _and CO_2 _ranged from 3.1 to 15.4 l/minute and from 3.4 to 15.1 l/minute, respectively. CO_1 _and CO_2 _were closely correlated (*r*^2 ^= 0.99), with mean bias (± SD) of 0.20 ± 0.30 l/minute and percentage error of 7% (Figure 3). GEDV_1 _and GEDV_2 _ranged from 701 to 1,629 ml and from 774 to 1,645 ml. GEDV_1 _and GEDV_2 _were closely correlated (*r*^2 ^= 0.79), with mean bias of -11 ± 80 ml and percentage error of 14% (Figure 4). EVLW_1 _and EVLW_2 _ranged from 507 to 2,379 ml and from 495 to 2,222 ml. EVLW_1 _and EVLW_2 _were closely correlated (*r*^2 ^= 0.97), with mean bias of -5 ± 72 ml and percentage error of 15% (Figure 5).

**Figure 6:**
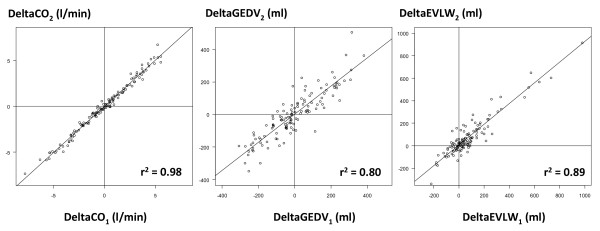
**Correlations between changes in hemodynamic parameters between the two measurement methods**. Correlations between changes in cardiac output (CO), changes in global end-diastolic volume (GEDV) and changes in extravascular lung water (EVLW) measured by the PiCCO™ system (CO_1_, GEDV_1 _and EVLW_1_) and by the VolumeView™ system (CO_2_, GEDV_2 _and EVLW_2_).

Changes in CO_2_, GEDV_2_, and EVLW_2 _were closely correlated with changes in CO_1_, GEDV_1_, and EVLW_1_, respectively (Figure 6).

The effects of each intervention are summarized in Table 2. Inotropic stimulation (DOBU) was achieved by administering an average 23 μg/kg/minute dose of dobutamine, hypovolemia (HYPO) by an average 1.2 l controlled hemorrhage, and hypervolemia (HYPER) by the average infusion of 4.5 l serum saline and 1.5 l gelatin. Both GEDV_1 _and GEDV_2 _decreased significantly during bleeding and increased significantly after blood restitution and fluid loading (Table 2). EVLW_1 _and EVLW_2 _increased slightly but significantly during fluid overload and dramatically (+110%) during ALI (Table 2). At each stage, values measured with the new VolumeView™ and with the current PiCCO™ method were comparable (Table 2).

**Table 2:** Transpulmonary thermodilution parameters over the study period

	BASE1	DOBU	BASE2	HYPO	BASE3	HYPER	ALI
CO_1 _(l/min)	7.5 ± 0.9, 7.6 (6.9 to 8.2)	10.8 ± 1.4*, 10.9 (10.0 to 11.5)	7.5 ± 0.7, 7.4 (7.2 to 7.8)	4.7 ± 0.3*, 4.9 (4.5 to 4.9)	7.9 ± 1.2, 7.7 (7.2 to 8.6)	11.7 ± 2.1*, 11.8 (10.1 to 13.1)	6.7 ± 3.3*, 5.2 (4.5 to 8.7)
CO_2 _(l/min)	7.6 ± 0.8, 7.9 (7.3 to 8.1)	11.0 ± 1.6*, 11.0 (10.1 to 11.7)	7.6 ± 0.8, 7.3 (7.1 to 8.1)	4.8 ± 0.2*, 4.9 (4.6 to 4.9)	8.0 ± 1.2, 8.0 (7.3 to 8.7)	12.0 ± 2.1*, 11.8 (10.5 to 13.5)	6.9 ± 3.4*, 5.7 (4.6 to 9.0)
GEDV_1 _(ml)	1,077 ± 149, 1,116 (953 to 1,171)	1,059 ± 134, 1,001 (958 to 1,167)	1,110 ± 147, 1,139 (990 to 1,230)	925 ± 84*, 943 (885 to 977)	1,173 ± 120, 1,164 (1,102 to 1,240)	1,326 ± 140*, 1,288 (1,245 to 1,440)	1,070 ± 191*, 1,144 (929 to 1,170)
GEDV_2 _(ml)	1,052 ± 94, 1,040 (1,009 to 1,076)	1,023 ± 102, 1,038 (942 to 1,056)	1,093 ± 124, 1,117 (1,000 to 1,177)	931 ± 66*, 937 (911 to 978)	1,153 ± 100, 1,157 (1089 to 1,214)	1,299 ± 162*, 1,322 (1,218 to 1,363)	1,089 ± 174*, 1,118 (976 to 1,194)
EVLW_1 _(ml)	622 ± 86, 627 (558 to 684)	691 ± 112, 650 (631 to 723)**	653 ± 106, 639 (577 to 692)	609 ± 72*, 597 (549 to 675)	644 ± 82, 638 (563 to 710)	754 ± 117, 804 (654 to 858)**	1,587 ± 380, 1,609 (1,305 to 1,711)**
EVLW_2 _(ml)	621 ± 82, 613 (552 to 683)	642 ± 68, 628 (596 to 666)	635 ± 85, 619 (592 to 678)	624 ± 68, 626 (580 to 679)	624 ± 80, 587 (567 to 710)	749 ± 128*, 750 (654 to 823)	1,571 ± 335*, 1,580 (1,374 to 1,752)

Results are expressed as the mean ± standard deviation, median (interquartile range). CO, cardiac output; GEDV, global end-diastolic volume; EVLW, extravascular lung water; subscript 1, current method (PiCCO™; Pulsion); subscript 2, new method (VolumeView™; Edwards); BASE, baseline; DOBU, dobutamine infusion; HYPO, hypovolemia induced by bleeding; HYPER, hypervolemia induced by volume loading; ALI, acute lung injury induced by oleic acid. *P < 0.01 (DOBU vs. BASE1 or HYPO vs. BASE2 or HYPER vs. BASE3 or ALI vs. HYPER); normal distribution, paired *t *test. **P < 0.01 (DOBU vs. BASE1 or HYPER vs. BASE3 or ALI vs. HYPER); abnormal distribution, nonparametric paired Wilcoxon signed-rank test. At each stage, values measured with the new VolumeView™ and with the current PiCCO**™ **method were comparable.

## Discussion

In animals, and over a wide range of values, the present study demonstrates that GEDV and EVLW derived from the new VolumeView™ method and from the current PiCCO™ method are interchangeable.

Both methods derive CO from the TPTD curve using the Stewart-Hamilton principles and the same equation [4] so, not surprisingly, the agreement was extremely good with a percentage error of 7%, far below the clinically acceptable threshold value of 30% proposed by Critchley and Critchley [13].

In contrast, GEDV was derived from two different equations. The PiCCO™ equation is based on time characteristics of the TPTD curve (mean transit time of the cold indicator and exponential downslope time) while the new VolumeView™ equation additionally relies on the ascending and descending slopes of the dilution curve (Figure 1). The present results show that both methods are interchangeable to assess GEDV even when significant changes in cardiac preload are induced by bleeding and fluid loading. They also confirm that GEDV is not affected by dobutamine-induced changes in CO, and hence that there is no mathematical coupling between both parameters [3,5]. The GEDV has been shown to be a reliable indicator of cardiac preload [5], varying in the same direction as echocardiographic preload indices [6]. A goal-directed strategy based on the optimization of GEDV has been shown to be useful to improve the postoperative outcome of cardiac surgical patients [15].

Both methods were also interchangeable for the assessment of EVLW; not only during slight modifications induced by fluid overload, but also during dramatic increases related to capillary leak as those observed during the ALI phase (Table 2). Assessing EVLW may be useful for clinicians treating patients with ALI or left ventricular failure [16]. EVLW has been shown to be more sensitive and specific than chest X-ray and ALI criteria to diagnose pulmonary edema [17,18]. EVLW is also a prognostic parameter since it has repeatedly been shown to be correlated with mortality in patients with ALI as well as in the general intensive care unit population [19-22]. Moreover, it has been suggested in critically ill patients that goal-directed strategies based on the measurement of EVLW may be associated with a decrease in the duration of mechanical ventilation and length of hospital stay [15,23,24].

Surprisingly, EVLW_1 _increased slightly but significantly during dobutamine infusion and decreased slightly but significantly during bleeding, while EVLW_2 _did not change (Table 2). From a pathophysiological point of view, no change in lung water is expected during inotropic stimulation or hypovolemia, particularly over such a short period of time [25]. Since our study was not designed to compare the PiCCO™ method and the VolumeView™ method with a third reference method (such as gravimetry), however, we cannot draw any definitive conclusions regarding the superiority of one method over the other.

Our study also confirms the very good reproducibility of TPTD measurements. These findings are in line with previous studies [5,26] reporting reproducibility of CO, GEDV and EVLW of 4 to 7%, 5 to 8% and 11%, respectively.

## Study limitations

The gravimetric method in animals and the double indicator (cold green dye) dilution method in humans are considered gold standard methods to quantify EVLW [9,10]. The goal of the present study was to compare the new VolumeView™ system with the TPTD system currently in clinical use - this is why the PiCCO™ system has been selected as the reference method in our study. A clinical validation is necessary to investigate whether the new VolumeView™ system is also comparable with the PiCCO™ system in critically ill patients. The new VolumeView™ algorithm was originally developed to decrease the sensitivity of TPTD to recirculation and thermal baseline drifts. The present study was not designed to investigate this potential advantage over the existing TPTD technology, but instead to ensure that the new VolumeView™ system and the PiCCO™ system are interchangeable in clinical-like conditions where CO, blood volume and lung water vary significantly. Further studies are therefore required to compare both systems in situations where technical (thermal baseline drift) or other clinical challenges (for example, valvular regurgitation-induced recirculation) are encountered.

## Conclusions

In animals, and over a very wide range of values, the new TPTD VolumeView™ system is comparable with the current PiCCO™ system to assess CO, GEDV and EVLW during inotropic stimulation, acute hemorrhage, fluid overload and severe acute lung injury.

## Key messages

• TPTD is increasingly used for hemodynamic evaluations in critically ill patients.

• The TPTD method currently in clinical use and implemented in the PiCCO™ system (Pulsion Medical Systems) is based on mathematical models described in the 1950 s.

• A new and original method has recently been developed to derive GEDV and EVLW from a TPTD curve (VolumeView™; Edwards Lifesciences).

• In animals, and over a very wide range of values, the new transpulmonary thermodilution VolumeView™ system is comparable with the current PiCCO™ system to assess CO, GEDV and EVLW during inotropic stimulation, acute hemorrhage, fluid overload and severe acute lung injury.

## Abbreviations

ALI: acute lung injury induced by oleic acid; CO: cardiac output; CO_1_: cardiac output measured by PiCCO_2_™; CO_2_: cardiac output measured by EV1000; DOBU: dobutamine infusion; GEDV: global end-diastolic volume; GEDV_1_: global end-diastolic volume measured by PiCCO_2_™; GEDV_2_: global end-diastolic volume measured by EV1000; EVLW: extravascular lung water; EVLW_1_: extravascular lung water measured by PiCCO_2_™; EVLW_2_: extravascular lung water measured by EV1000; HYPO: hypovolemia induced by bleeding; HYPER: hypervolemia induced by volume loading; MAP: mean arterial pressure; SD: standard deviation; TPTD: transpulmonary thermodilution.

## Competing interests

KB received consultant fees from Edwards LifeSciences. FM is a director at Edwards Lifesciences and is coinventor on transpulmonary thermodilution patents (US2005267378, US2007282213, WO2009049872). RG and NS have no potential conflicts of interest to declare.

## Authors' contributions

KB and FM designed the study and wrote the article. KB was responsible for data collection and data analysis, with the help of RG and NS. All authors reviewed and approved the final manuscript.
